# Fast Method for Liquid Crystal Cell Spatial Variations Estimation Based on Modeling the Spectral Transmission

**DOI:** 10.3390/s19183874

**Published:** 2019-09-08

**Authors:** Shauli Shmilovich, Liat Revah, Yaniv Oiknine, Isaac August, Ibrahim Abdulhalim, Adrian Stern

**Affiliations:** 1 Department of Electrical and Computer Engineering, School of Electrical and Computer Engineering, Ben-Gurion University of the Negev, P.O.B. 653, Beer-Sheva 8410501, Israel; 2 Department of Electro-Optics and Photonics Engineering, School of Electrical and Computer Engineering, Ben-Gurion University of the Negev, P.O.B. 653, Beer-Sheva 8410501, Israel

**Keywords:** liquid crystal, spectral transmission, sensor calibration, spatial variations, spatial non-uniformity, spatial estimation algorithm

## Abstract

Liquid crystal phase retarders are utilized by photonic devices and imaging systems for various applications, such as tunable filtering, light modulation, polarimetric imaging, remote sensing and quality inspection. Due to technical difficulties in the manufacturing process, these phase retarders may suffer from spatial non-uniformities, which degrade the performance of the systems. These non-uniformities can be characterized by measuring the spectral transmission at each voltage and each point on the liquid crystal cell, which is time consuming. In this work, we present a new fast and simple method for measuring and computationally estimating the spatial variations of a liquid crystal phase retarder with planar alignment. The method is based on measuring the spectral transmission of the phase retarder at several spatial locations and estimating it at others. The experimental results show that the method provides an accurate spatial description of the phase retarder and can be employed for calibrating relevant systems.

## 1. Introduction

Liquid crystal phase retarders, whether in single pixel or pixelated formats, besides their traditional use in displays, are used in many non-display applications [1] such as photonic devices [2] and imaging systems of different purposes. These include systems for multidimensional imaging [3], remote sensing from space [4], biomedical imaging [5], spectral imaging of paintings [6], and ellipso-polarimetric imaging [7,8]. Achieving a spatially uniform liquid crystal cell (LCC) remains a fabrication challenge [9], and gap variations appear throughout the cell [10]. As the LCC’s retardance and spectral transmission (ST) depend on the cell gap, they suffer from spatial variations as well, affecting the performance of the systems utilizing the LCC. Therefore, it is important to experimentally measure the spatial variations of a LCC. Several methods for performing such measurements have been proposed in previous studies. For instance, spatial variations can be detected by means of polarimetric imaging, using monochromatic illumination [10]. While this method enables a quick detection of the spatial variations, it allows viewing their effect on the LCC’s retardance and ST at a single wavelength solely. Therefore, it does not provide a full spectral and spatial characterization of the LCC. An additional method is to translate the LCC to perform measurements at different spatial locations and use prior information about the properties of the LCC for spatial variations estimation [11]. Another approach, given that the birefringence of the liquid crystal material is known, is to measure the cell gap at a single location by using a rotating polarizer method [12], or a rotating quarter-wave retarder method [13]. Then, by measuring the gap at many locations, a full spatial characterization of the cell could be obtained. The downside to this approach is that it demands rotating optical components in order to achieve the spatial characterization and that performing such measurements at many locations could be very time consuming. In addition, these types of measurements are usually performed with no voltage applied to the LCC, while its retardation spectrum is voltage-dependent. Thus, they do not account for possible deviations from the ideal liquid crystal switching model with the application of an electric field, which may occur due to non-idealities in the cell’s shape and structure. For example, due to non-uniformities on the substrates, different absorptions at different wavelengths or existence of defects could occur. Therefore, performing measurements with different voltages applied to the LCC is crucial for obtaining a realistic and accurate characterization. As the research presented in this paper was primarily motivated for the development of a new calibration process for the LCC-based Compressive Sensing Miniature Ultra-Spectral Imaging (CS-MUSI) system [14,15,16,17], it had several distinct objectives. Firstly, to develop a method to measure and estimate the ST of the system as a whole, that is, without changing the state and structure of the system [12,13], e.g., without rotating any of its components. Secondly, to characterize the system’s ST spatially, at a desired wavelengths range and with different voltages applied to its LCC. This means that the ST’s spatial- and voltage-dependent variations caused by any kind of non-ideality should be incorporated into the ST’s characterization and that the detection of spatial non-uniformities at a single wavelength [10] is not sufficient. Lastly, the method needs to be fast and not time consuming, measuring the ST at every wavelength, voltage and location of the LCC. Additionally, it should be noted that one of the advantages of the presented method, is that in contrast to Refs. [11,12,13], it does not rely on prior information regarding the birefringence of the LCC in order to perform the ST spatial variations estimation.

**Figure 1:**
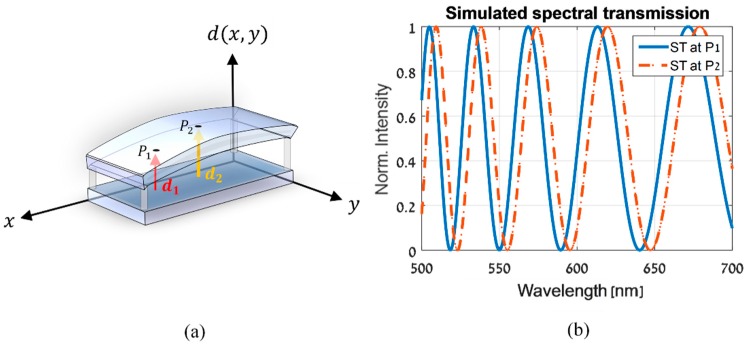
(**a**) A demonstration of a spatially non-uniform LCC. The cell gap d(x,y) changes spatially, as can be seen by comparing $d_{1}$ and $d_{2}$ at points P1 and P2. (**b**) A comparison between simulated STs at points P1 and P2 which shows how they differ due to the spatial variations of the LCC, when an arbitrary voltage is applied to it.

The previously mentioned CS-MUSI system relies on the framework of compressive sensing (CS) [18]. It enables the acquisition of hyperspectral images by an order of magnitude fewer measurements compared to classical hyperspectral imaging systems. In order to do so, it utilizes a thick LCC phase retarder as a compact wide band spectral modulator. As thick LCCs with a cell gap of approximately 50 μm are not commercially available, the LCC was manufactured by the liquid crystals’ group of Prof. Abdulhalim at Ben-Gurion University. It consists of a layer of liquid crystal material, which was inserted between two flat glass plates coated with Indium Tin Oxide (ITO). The glass plates were spin coated with a polymer-alignment layer, and glass spacers were applied on the edges to determine the nominal thickness, which defines the cell gap. Due to differences between the nominal and real sizes of the glass spacers and the non-ideal flatness of the glass plates, the LCC is spatially non-uniform (Figure 1a). As a result, it has a spatially varying ST, as demonstrated in Figure 1b. Since knowing the exact ST of the system is crucial for CS reconstruction algorithms, we have developed a new calibration process in this work, which consists of a method for measuring the ST and estimating its spatial dependency.

## 2. Optical Calibration Setup

**Figure 2:**
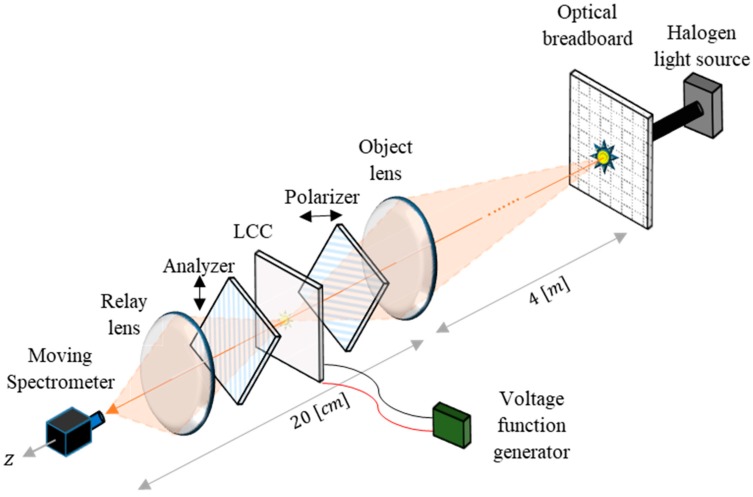
The optical calibration setup scheme. A point source is generated by directing halogen light with an optical fiber to specific locations on the optical breadboard placed perpendicular to the optical axis. An objective lens forms an image of the light source on the LCC, which is located between a crossed polarizer and analyzer. A relay lens maps the image 1:1 to the spectrometer plane. The voltage applied to the LCC is adjustable using the voltage function generator.

The scheme of the optical calibration setup, which was designed in order to enable the measurement of the CS-MUSI system’s ST, is shown in Figure 2. It consists of an objective lens which forms an image on the system’s LCC. The LCC is placed between a polarizer and analyzer which are linear, crossed and oriented at a 45-degrees angle with respect to the optical axis of the LCC. The light transmitted through the polarizer-LCC-analyzer subsystem is captured by a moving commercial grating spectrometer, in a plane that is optically conjugated to the LCC using a 1:1 relay lens. Different voltages are applied to the LCC using a function generator of sine waveform voltages of different amplitudes in the range 0~10 V. The imaged object is a distant halogen point light source, which can be placed at different locations by using an optical stage. Instead of a stage, we used an available optical breadboard which was placed perpendicular to the optical axis. By doing so, the light transmitted through the LCC at different spatial locations can be measured at a desired wavelength range using the moving spectrometer and at desired voltages using the function generator. Then, by using the method we propose in this paper, the ST of the system can be modeled and estimated at unmeasured locations of the LCC.

This optical calibration setup is based on the experimental setup form of the CS-MUSI system [14]. The difference between them is that the sensor array of the CS-MUSI system is replaced by the moving grating spectrometer.

## 3. Spectral Transmission Parametric Modeling and Spatial Estimation Method

### 3.1. Spectral Transmission Parametric Modeling

In order to estimate the spatial variations of the LCC and the ST, a parametric model of the ST is derived. For a spatially uniform LCC with planar alignment, the normalized ST intensity of the previously-mentioned polarizer-LCC-analyzer subsystem, which will be referred to as the ST of the LCC, is given by [19]

(1) $$i\left(\lambda ,V\right)=\frac{1}{2}-\frac{1}{2}\cos\left(\phi \left(\lambda ,V\right)\right).$$

Here, λ is the wavelength, V is the voltage applied to the LCC, and ϕ(λ,V) denotes the retardance of the LCC retarder, which reads as

(2) $$\begin{array}{ccc} \phi \left(\lambda ,V\right)=\frac{2\pi d}{\lambda }\Delta n\left(\lambda ,V\right) & , & \Delta n\left(\lambda ,V\right)=n_{eff}\left(\lambda ,\theta \left(V\right)\right)-n_{o}\left(\lambda \right). \end{array}$$

The cell gap is denoted by d, and Δn(λ,V) is the effective birefringence of the LCC, where $n_{eff}\left(\lambda ,\theta \left(V\right)\right)$ and $n_{o}\left(\lambda \right)$ are the effective extraordinary and ordinary refractive indices, respectively. When a voltage is applied to the LCC, it affects the molecular tilt angle, θ(V), of the LCC’s director. θ(V) then stabilizes on a value in the range of 0°–90°. When the value of θ(V) is changed, it alters the effective extraordinary refractive index, which is given by [20,21]

(3) $$n_{eff}\left(\lambda ,\theta \left(V\right)\right)=\frac{n_{o}\left(\lambda \right)n_{e}\left(\lambda \right)}{\sqrt{n_{o}^{2}\left(\lambda \right)\cos^{2}\left(\theta \left(V\right)\right)+n_{e}^{2}\left(\lambda \right)\sin^{2}\left(\theta \left(V\right)\right)}},$$

where $n_{e}\left(\lambda \right)$ refers to the extraordinary refractive index of the LCC. At zero voltage, and therefore ideally at zero tilt, $n_{eff}\left(\lambda ,\theta \left(V\right)\right)$ is equal to $n_{e}\left(\lambda \right)$, and the effective birefringence is therefore $\Delta n\left(\lambda ,0\right)=n_{e}\left(\lambda \right)-n_{o}\left(\lambda \right)$. Equation (2) can then be written as

(4) $$\begin{array}{ccc} \phi \left(\lambda ,V\right)=\frac{2\pi d}{\lambda }g\left(V\right)\Delta n\left(\lambda ,0\right) & , & g(V)\approx g\left(\lambda ,V\right)≜\frac{\Delta n\left(\lambda ,V\right)}{\Delta n\left(\lambda ,0\right)} \end{array}=\frac{n_{eff}\left(\lambda ,\theta \left(V\right)\right)-n_{o}\left(\lambda \right)}{n_{e}\left(\lambda \right)-n_{o}\left(\lambda \right)}.$$

For practical values of ordinary and extraordinary refractive indices, the normalized effective birefringence function, g(λ,V), is known to be approximately non-dependent on the wavelength [10,20]. It will therefore be denoted as g(V). In addition, from the Cauchy dispersion equation, we know that each of the ordinary and extraordinary refractive indices, and hence, the effective birefringence at zero voltage, decreases as the wavelength increases [22,23,24,25]. By using the first three terms of the Cauchy dispersion equation, the effective birefringence at zero voltage can be approximated by

(5) $$\Delta n\left(\lambda ,0\right)=a+\frac{b}{\lambda^{2}}+\frac{c}{\lambda^{4}}.$$

Higher order terms of the Cauchy dispersion equation could also be incorporated, but excellent results were obtained using the first three terms solely, and the higher terms were omitted for the simplicity of the model. The spatial non-uniformity of the LCC is included in the parametric model by denoting the cell gap d as d(x,y). Note that the x and y axes are defined in Figure 1a with respect to the LCC. Using the new notation for the spatially-dependent cell gap, and from Equations (4) and (5), it follows that the retardance is voltage, wavelength- and spatially-dependent, and can be written as

(6) $$\phi \left(\lambda ,V;x,y\right)=\frac{2\pi d\left(x,y\right)}{\lambda }g\left(V\right)\Delta n\left(\lambda ,0\right)=\frac{A\left(V;x,y\right)}{\lambda }+\frac{B\left(V;x,y\right)}{\lambda^{3}}+\frac{C\left(V;x,y\right)}{\lambda^{5}},$$

where the values of the functions A(⋅), B(⋅), and C(⋅) depend on the location on the LCC, and the voltage applied to the cell. The terms A(⋅), B(⋅), and C(⋅) are given by

(7) $$\begin{array}{c} A\left(V;x,y\right)=2\pi ag\left(V\right)d\left(x,y\right),\\ B\left(V;x,y\right)=2\pi bg\left(V\right)d\left(x,y\right),\\ C\left(V;x,y\right)=2\pi cg\left(V\right)d\left(x,y\right). \end{array}$$

According to Equation (1), the intensity of the ST exhibits a full modulation depth, that is, it reaches the values 0 and 1 at all of its minima and maxima. This describes the ideal case, when achieving the uniform anchoring strength of the LCC molecules and a uniform pre-tilt angle. However, in practice, the anchoring strength and the pre-tilt angle are not uniform throughout the cell, and the measured ST does not achieve a full modulation depth. There are also many other additional non-uniformities which could have similar effects on the ST. These include non-idealities of the uniformity of the polarizers, glass plates, absorption of the LCC and the ITO, or defects in the liquid crystal layer. In order to take these contrast variations of the retardation spectrum into consideration and incorporate them into our parametric model, we include a variable envelope function, Env(λ,V). The envelope function is wavelength- and voltage-dependent, and inserted into our parametric model via multiplication with the cosine term in Equation (1). Then, from Equations (1) and (6), the normalized ST intensity can be written as

(8) $$i\left(\lambda ,V;x,y\right)=\frac{1}{2}-Env\left(\lambda ,V\right)\cos\left(\frac{A\left(V;x,y\right)}{\lambda }+\frac{B\left(V;x,y\right)}{\lambda^{3}}+\frac{C\left(V;x,y\right)}{\lambda^{5}}\right),$$

**Figure 3:**
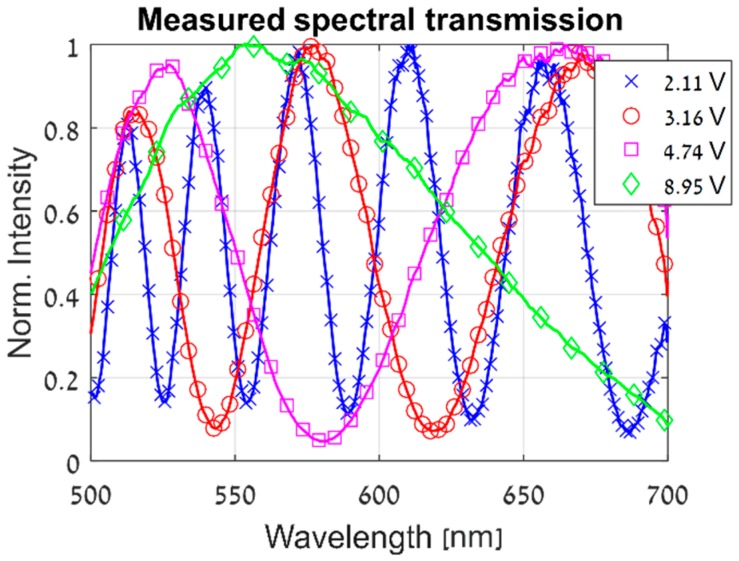
The ST of the CS-MUSI system’s LCC, measured at the same location and with four different voltages applied to the LCC.

The model in Equation (8) enables us to parametrically describe the voltage, spatial and wavelength dependencies of the ST of the LCC, while considering its non-idealities. It allows to estimate the spatial variations of the entire cell by measuring the ST at several locations. Figure 3 demonstrates the dependence of the CS-MUSI system’s ST on both the wavelength and the voltage applied to the LCC, as modeled in Equation (8). The spatial dependency of the ST was previously demonstrated in Figure 1.

### 3.2. Spatial Estimation

**Figure 4:**
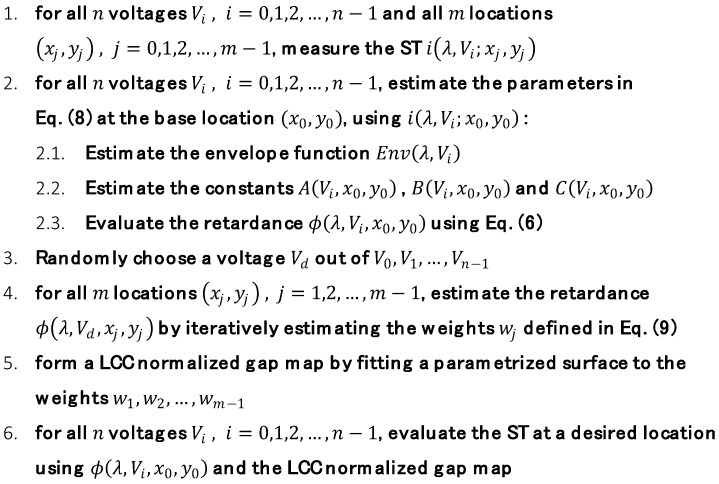
The spatial estimation algorithm. The ST is measured at several locations and voltages. It is then parametrically modeled at the base location $\left(x_{0},y_{0}\right)$, and its spatial variations are estimated. This allows to evaluate the ST at a desired location.

**Figure 5:**
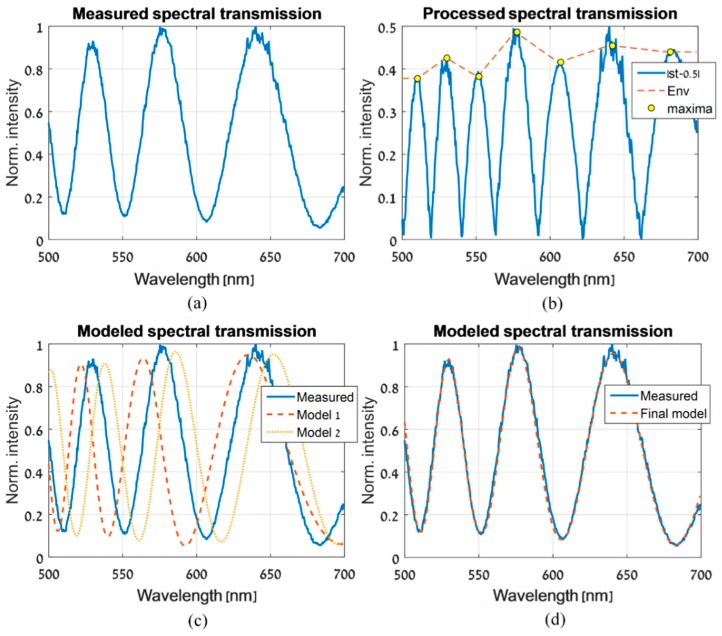
The estimation process of the ST model parameters, which are introduced in Equation (8), are performed at the randomly chosen base location, $\left(x_{0},y_{0}\right)$. (**a**) The normalized ST, measured at $\left(x_{0},y_{0}\right)$, with a randomly chosen voltage, $V_{i}$, applied to the LCC. (**b**) The ST, after subtracting 0.5 and calculating its absolute value. The maxima points and the calculated $Env\left(\lambda ,V_{i}\right)$ function are shown. (**c**) Two models are shown, which were obtained using Equation (8) and by gridding $A\left(V_{i};x_{0},y_{0}\right)$, $B\left(V_{i};x_{0},y_{0}\right)$ and $C\left(V_{i};x_{0},y_{0}\right)$. These models are a poor description of the measured ST, and will not be chosen as the final ST model. (**d**) The final model and the measured ST.

The spatial estimation algorithm is described in Figure 4. It begins by measuring the ST of the LCC at some m locations, sparsely chosen over the cell. At each location, the ST is measured at n different voltages that are applied to the LCC, and are denoted as $V_{i}$ where i=0,…,n−1. One randomly chosen location is defined as the base location, and its spatial coordinates are denoted as $\left(x_{0},y_{0}\right)$. The next step is to estimate the parameters of the model given in Equation (8) at the chosen base location. This step is demonstrated in Figure 5. The envelope function, $Env\left(\lambda ,V_{i}\right)$, is estimated at each applied voltage $V_{i}$, using the measured ST versus wavelength data (Figure 5a). Its estimation is obtained by subtracting the oscillating measured ST from the bias value, calculating its absolute value, finding the intensity values at the maxima and performing a linear interpolation to obtain the values at all of the desired wavelength range (Figure 5b). Next, the constants $A\left(V_{i};x_{0},y_{0}\right)$, $B\left(V_{i};x_{0},y_{0}\right)$ and $C\left(V_{i};x_{0},y_{0}\right)$ are estimated for each voltage $V_{i}$. This is achieved by creating a three-dimensional grid of evenly spaced values with $A\left(V_{i};x_{0},y_{0}\right)$, $B\left(V_{i};x_{0},y_{0}\right)$ and $C\left(V_{i};x_{0},y_{0}\right)$ as its axes. By running through all the points in the grid and seeking maximum correlation between the modeled ST in Equation (8) and the measured ST (Figure 5c,d), the estimated values of $A\left(V_{i};x_{0},y_{0}\right)$, $B\left(V_{i};x_{0},y_{0}\right)$ and $C\left(V_{i};x_{0},y_{0}\right)$ are determined. This completes the estimation of all the parameters in Equation (8) at the base location. In addition, using Equation (6) and the estimated values of $A\left(V_{i};x_{0},y_{0}\right)$, $B\left(V_{i};x_{0},y_{0}\right)$ and $C\left(V_{i};x_{0},y_{0}\right)$, we obtain an estimation of the retardance $\phi \left(\lambda ,V_{i};x_{0},y_{0}\right)$ for each voltage $V_{i}$.

At this stage, we have obtained a parametric model, $i\left(\lambda ,V;x_{0},y_{0}\right)$, which describes the measured ST at the base location, at the desired applied voltages and wavelengths range. We are now interested in using it to help us estimate the spatial variations of the ST and LCC. We start by denoting the spatial coordinates of the locations in which we have measured the ST as $\left(x_{j},y_{j}\right)$ with j=0,…,m−1, where m is the total number of measured locations. Note that at the base location, j is 0. Our next step is to obtain a parametric model of the ST for each of the remaining m−1 locations, at a single voltage $V_{d}$ (the subscript “d” indicates that d(x,y) will be estimated at this voltage). This is achieved by randomly choosing the value of $V_{d}$ from the n applied voltages, and iteratively estimating the retardance $\phi \left(\lambda ,V_{d};x_{j},y_{j}\right)$ at the remaining m−1 locations, using the retardance $\phi \left(\lambda ,V_{d};x_{0},y_{0}\right)$ at the base location. Under the assumption that the effective birefringence does not vary spatially, Equation (6) implies that the value of the retardance at different locations, differs solely due to different d(x,y) values. Therefore, at each of the remaining locations, $\phi \left(\lambda ,V_{d};x_{j},y_{j}\right)$ is initialized by the product of $\phi \left(\lambda ,V_{d};x_{0},y_{0}\right)$ and a weight $w_{j}$. This gives us

(9) $$\begin{array}{ccc} \phi \left(\lambda ,V_{d};x_{j},y_{j}\right)=\phi \left(\lambda ,V_{d};x_{0},y_{0}\right)w_{j}, & & \forall j=1,\ldots ,m-1 \end{array}.$$

Each weight $w_{j}$ is initialized to the value 1 and changed iteratively seeking maximum correlation between the parametric model of the ST and the measured ST at each location, both at the voltage $V_{d}$. As the final value of $w_{j}$ is obtained for each location, we proceed by dividing the iteratively determined retardance at each location, by that of the base location. From Equations (6) and (9), we obtain

(10) $$\begin{array}{ccc} \frac{\phi \left(\lambda ,V_{d};x_{j},y_{j}\right)}{\phi \left(\lambda ,V_{d};x_{0},y_{0}\right)}=w_{j}=\frac{d\left(x_{j},y_{j}\right)}{d\left(x_{0},y_{0}\right)}, & & \forall j=1,\ldots ,m-1 \end{array},$$

meaning that the weights $w_{j}$ are the values of the LCC gaps at all m−1 locations, normalized by the gap at the base location. Thus, the weights can be used to form a normalized gap map that estimates the spatial variations of the LCC and ST. This is achieved by fitting the values of the weights to a parametrized surface. For instance, a polynomial surface can be used. The degree of the polynomial surface is obtained by initially fitting to a surface with a first degree polynomial dependency for both x and y and gradually increasing the degree until reaching a minimal error while avoiding over-fitting. When the fitting process is complete, we obtain a normalized gap map $d\left(x,y\right)/d\left(x_{0},y_{0}\right)$ of the LCC. The parametric model of the LCC’s ST at the base location, along with the normalized gap map, provide us with a complete characterization of the LCC’s ST. In order to calculate the ST at a certain location, voltage and desired wavelength range, the parametric model of the base location at that voltage and wavelength range is chosen, and its retardance is multiplied by the value of the normalized gap map at that location. Equations (9) and (10) imply that the result of that product is the retardance at the desired location. Having estimated Env(λ,V) at the base location, we have all the parameters needed to calculate the ST at the wanted location, voltage, and wavelength range by using Equation (8).

## 4. Results

In this section, we demonstrate the performance of the proposed spatial estimation algorithm both on simulated and on real experimental data. In Section 4.1, a simulation of a spatially varying LCC is described, and the results obtained by applying the spatial estimation algorithm are presented. As in this section the ground truth of the geometrical profile of the LCC is simulated and therefore known, it is possible to compare it quantitatively to the estimated gap map obtained in the algorithm. In Section 4.2, we present the experimental estimation results achieved by applying the spatial estimation algorithm to the LCC of the CS-MUSI system.

### 4.1. Simulation

As an initial performance evaluation of the proposed spatial estimation algorithm, a LCC with a spatially-varying cell gap was simulated, and the estimation algorithm was applied to it. The geometrical profile chosen for the simulation was of curved glass plates, with cell dimensions of 1 cm × 1 cm × (45–50) μm, that is, with maximum cell gap variation of 5 μm. This was achieved by setting the spatially varying cell gap, d(x,y), as a second-degree polynomial surface, with different curvatures along the x and y axes. The analytic form of the simulated surface is given by

(11) $$\begin{array}{c} \begin{array}{ccc} d\left(x,y\right)=p_{00}+p_{10}y+p_{01}x+p_{20}y^{2}+p_{11}xy+p_{02}x^{2}, & & 0\leq x,y\leq 0.01\left[m\right], \end{array}\\ \left[p_{00},p_{10},p_{01},p_{20},p_{11},p_{02}\right]=\left[4.6,80,70,-8000,0,-6000\right]\cdot 10^{-5}. \end{array}$$

The liquid crystal substance chosen for the simulation was the nematic Merck BL036, and the Cauchy coefficients of its ordinary and extraordinary refractive indices, at zero voltage and visible wavelengths range, were taken from Ref. [26]. Using Equations (1)–(8), the simulated gap map (Equation (11)) and the Cauchy coefficients, the LCC’s ST could be calculated at the desired locations, applied voltages and wavelengths range.

**Figure 6:**
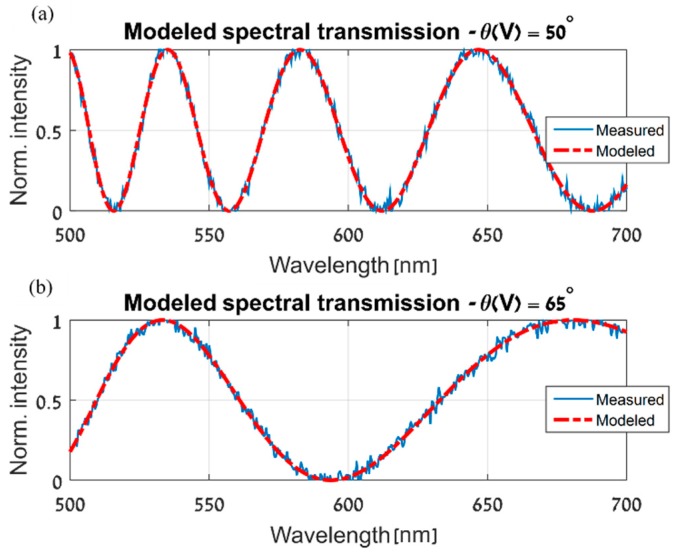
The parametric model as estimated at the chosen base location, $\left(x_{0},y_{0}\right)$, at two different molecular tilt angles, (**a**) 50° and (**b**) 65°.

With the simulated LCC at hand, the next step is to apply the proposed spatial estimation algorithm and evaluate its performance. As described in the spatial estimation section, the procedure begins by measuring the ST of the LCC at m different locations on the cell. To simulate such measurements, the ST was calculated at several locations on the simulated LCC, at the 500–700 nm wavelength range and with five random voltages applied to the cell. In order to simulate the application of different voltages to the simulated LCC, random values of tilt angles were used in Equation (3). In addition, using additive white Gaussian noise, a signal to noise ratio of 30 dB was set for the simulated ST measurements. Following the algorithm in Figure 4, a base location was randomly chosen out of the locations in which the simulated ST was measured. Then, a parametric model of the ST was estimated at the base location. This was performed for each of the applied voltages and at the relevant wavelength range. Figure 6 shows some examples of the successful outcome of the parametric modeling stage.

**Figure 7:**
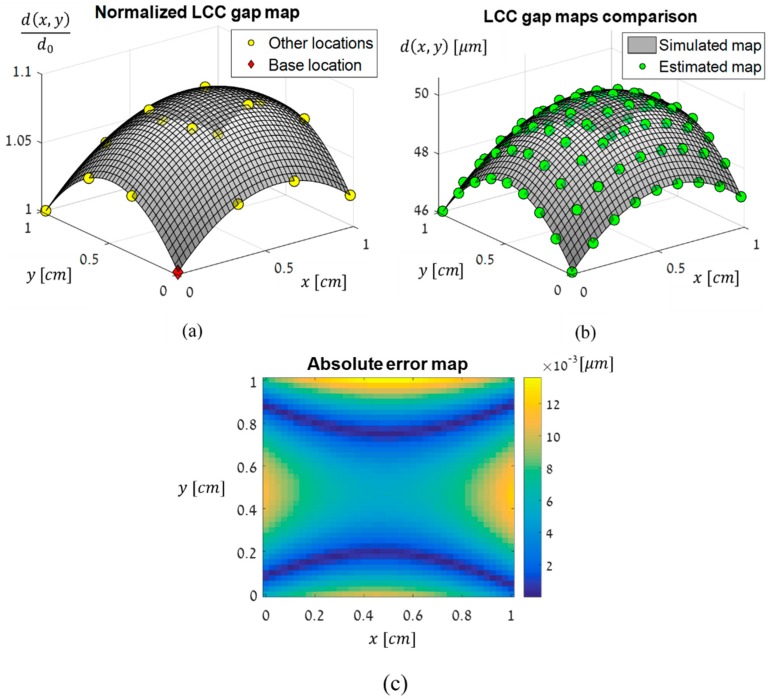
(**a**) The normalized estimated LCC gap map obtained in the simulation. The base location is marked with a red diamond, and the other locations with yellow circles. The map was formed at a single randomly chosen voltage. $d_{0}$ is the gap at the base location, and all other gap values are normalized by it. (**b**) The simulated and estimated gap maps layered one on top of the other. The simulated map is plotted as a gray surface, and the estimated map is plotted as green circles at evenly-spaced sampled locations. (**c**) The absolute error map which shows the estimation error across the cell (absolute value of the difference between the two maps in (**b**)).

In order to estimate the spatial variations of the simulated LCC and the ST, a normalized gap map was formed by finding the retardance at each measured location and dividing it by the retardance at the base location. All of the calculations were performed at a single randomly-selected voltage. By doing so, we obtained the normalized gap values and could fit them to a polynomial surface to form the full normalized gap map. In this simulation, fitting to a second-degree polynomial surface was sufficient. This was of course expected and in accordance with the simulated geometrical profile. The estimated normalized map and gap values are shown in Figure 7a. As the actual gap map was previously simulated, and is therefore known, it is possible to compare it to the estimated normalized gap map, when the latter is multiplied by the gap value at the base location. Figure 7b shows this comparison and that the maps are in excellent agreement. The absolute error map, which is obtained by subtracting the estimated gap map from the simulated gap map and calculating the absolute value of the result, is shown in Figure 7c. It can be seen that the error is small and in the order of a few nanometers across the cell, and therefore, causes negligible errors in the estimation of the ST.

In addition to the visual comparison, it is also possible to compare the parameters of the simulated polynomial gap map, given in Equation (11), to those obtained in the spatial estimation algorithm. By running the spatial estimation algorithm five times and using a different applied voltage for the gap map estimation in each run, we obtain

(12) $$\begin{array}{l} \left[{\hat{p}}_{00},{\hat{p}}_{10},{\hat{p}}_{01},{\hat{p}}_{20},{\hat{p}}_{11},{\hat{p}}_{02}\right]=\left[4.6,80.02,70.12,-7995.76,-4.74,-6006.96\right]\cdot 10^{-5},\\ \left[\Delta {\hat{p}}_{00},\Delta {\hat{p}}_{10},\Delta {\hat{p}}_{01},\Delta {\hat{p}}_{20},\Delta {\hat{p}}_{11},\Delta {\hat{p}}_{02}\right]=\left[4\cdot 10^{-4},0.37,0.16,33.31,8.57,13.06\right]\cdot 10^{-5}, \end{array}$$

which are the mean and standard deviation of each obtained estimated gap map parameter, respectively. It is evident that the average estimated values in Equation (12) are in excellent agreement with those in Equation (11), and any of the existing deviations cause negligible errors.

**Figure 8:**
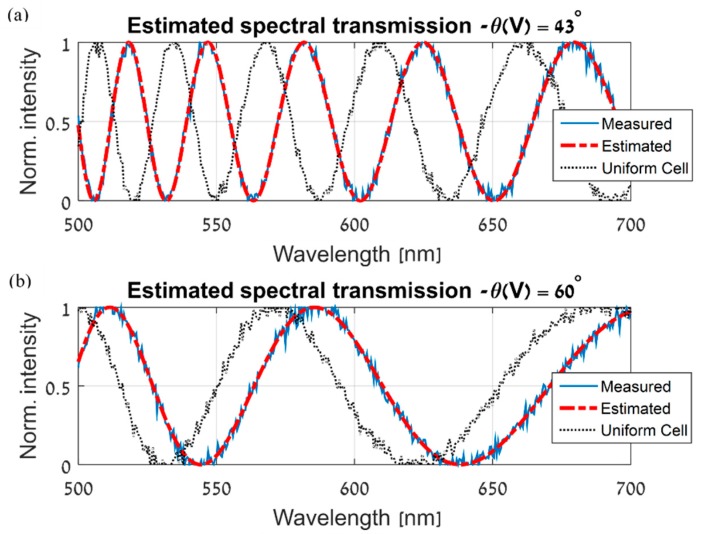
ST spatial estimation results at different voltages and locations on the simulated LCC. In each of (**a**,**b**), the continuous blue line is the measured ST at a specific location and voltage. The dashed red line is the estimated ST at the same location and voltage. Lastly, the dotted black line is the ST measured at the same voltage, but at the base location, demonstrating the error obtained without applying the proposed spatial estimation algorithm (that is, if a spatially uniform LCC is assumed, with a cell gap equal to the gap at the base location).

Using the estimated normalized gap map and the parametric model of the ST at the base location, the ST can be estimated at every location on the simulated LCC and at every measured voltage and wavelength. Figure 8 shows the final ST estimation results and the good agreement with the measured STs. In each of Figure 8a,b, three graphs are shown. The first is of the ST, measured at a specific location, voltage and the desired wavelength range. The second is the estimated ST at the same location, voltage and wavelength range. Lastly, the third graph is the ST, measured at the base location, at the same voltage and wavelength range. This third graph illustrates the importance of our work and the possible error describing the ST of the LCC, in case the cell is assumed to be spatially uniform, and its ST is measured only at a single location. It should be noted that in the procedure of obtaining each estimated ST in Figure 8, the corresponding ST, which was measured at that location, was excluded from the formation of the normalized gap map. Therefore, these results display excellent accuracy in the estimation of the ST at unmeasured locations on the LCC.

### 4.2. Experiment

**Figure 9:**
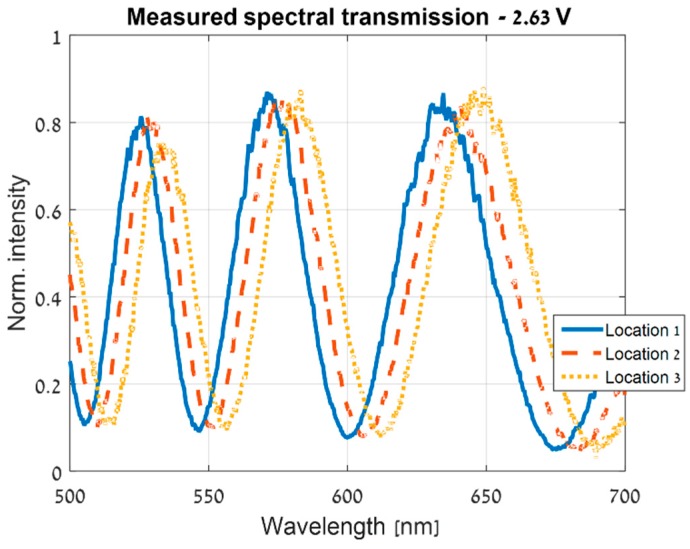
The ST measured with an applied voltage of 2.63 V, at three different locations on the CS-MUSI’s LCC. The non-uniform cell gap causes the ST to shift as the LCC’s retardance changes.

**Figure 10:**
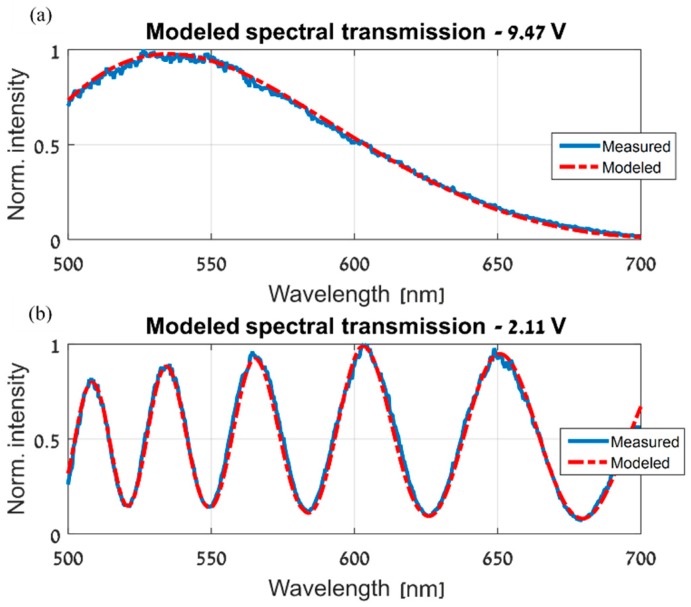
The parametric model as estimated at the chosen base location, $\left(x_{0},y_{0}\right)$, at two different voltages applied to the CS-MUSI’s LCC: (**a**) 9.47V and (**b**) 2.11V.

In this section, the spatial variations of the CS-MUSI’s LCC and its ST were estimated. Following the steps of the spatial estimation algorithm, as in the simulation section, the procedure begins by measuring the ST of the LCC. In our experiment, we measured the ST in the 500–700nm wavelength range, and at several locations and voltages. Figure 9 shows STs measured at the same voltage and wavelength range, but at different locations. It is clear that spatial variations exist in the LCC and significantly affect the ST. As in the simulation section, using the measured ST, a parametric model of the ST was then obtained at the randomly-chosen base location. This was done for each of the applied voltages and at the whole measured wavelength range. As shown in Figure 10, the measured ST and the achieved model are in good agreement.

**Figure 11:**
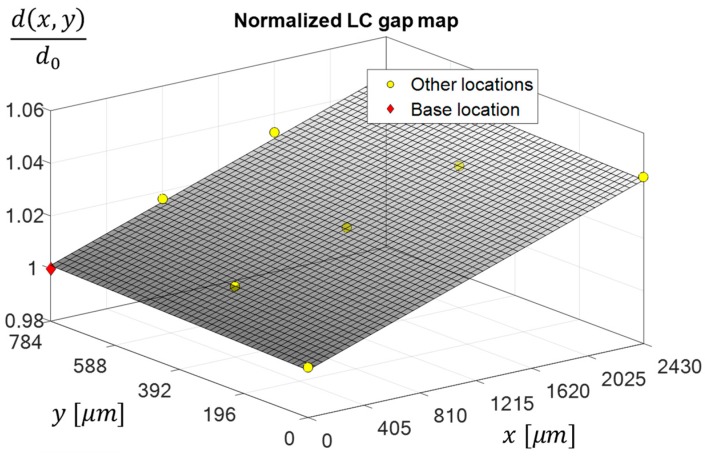
The normalized estimated LCC gap map obtained in the experiment. The base location is marked with a red diamond and the other locations with yellow circles. The map was formed at a single randomly-chosen voltage. $d_{0}$ is the gap at the base location, and all other gap values are normalized by it.

Proceeding to the next step of the algorithm, a normalized gap map was formed by finding the retardance at each measured location and dividing it by the retardance of the base location. All of the calculations were performed at a single randomly-selected voltage. By doing so, we obtained the normalized cell gap values and could fit them to a polynomial surface to form the full normalized gap map. In this experiment, fitting to a first-degree polynomial surface in both the x and y axes, was sufficient. The map is shown in Figure 11, and its spatial planar behavior can be seen clearly.

**Figure 12:**
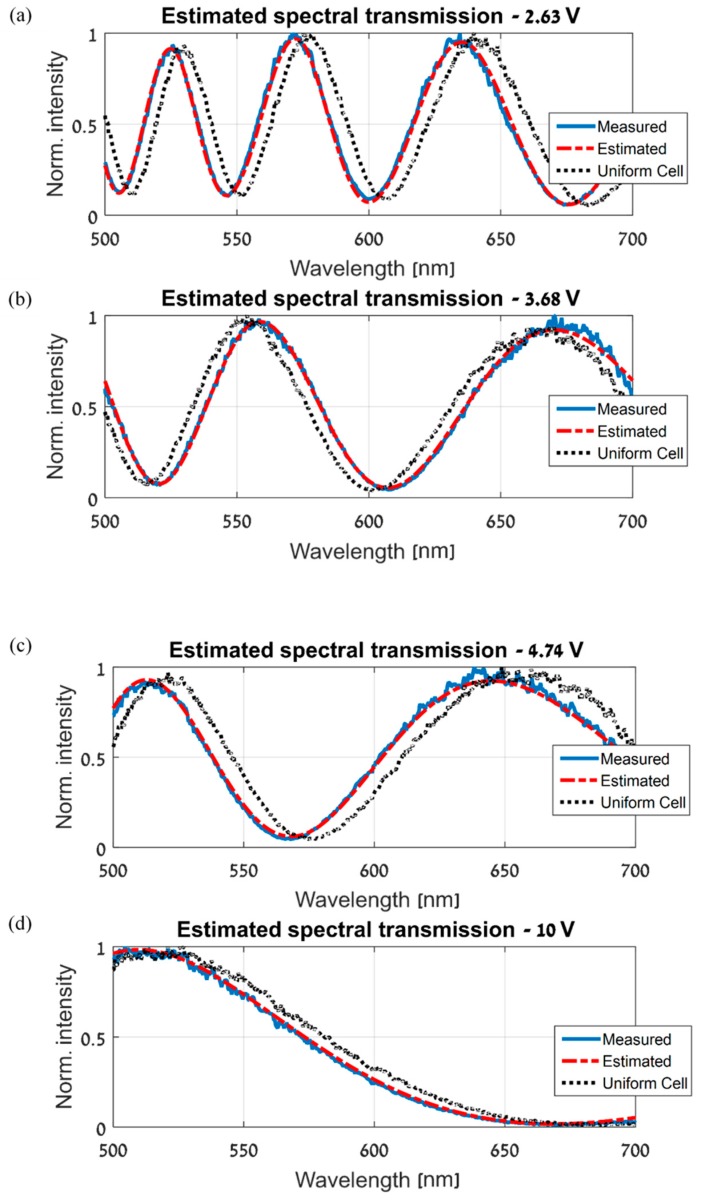
ST spatial estimation results at different voltages and locations on the CS-MUSI’s LCC. In each of (**a**–**d**), the continuous blue line is the measured ST at a specific location and voltage. The dashed red line is the estimated ST at the same location and voltage. Lastly, The dotted black line is the ST measured at the same voltage, but at the base location, demonstrating the error obtained without applying the proposed spatial estimation algorithm (that is, if a spatially uniform LCC is assumed, with a cell gap equal to the gap at the base location).

Using the normalized gap map and the parametric model of the ST that was measured at the base location, the ST can now be estimated at every location on the LCC, and at every measured voltage and wavelength. Figure 12 shows the final ST estimation results and the good agreement with the measured STs. The three graphs in each of Figure 12a–d are similar to the graphs shown and explained in Figure 8a,b.

## 5. Conclusions

In summary, in this paper, we have presented a simple method to estimate the spatial non-uniformity of a LCC with planar alignment and its effect on the ST of the cell. The method was then tested by simulation and an experiment. In the experiment, the CS-MUSI system and its polarizer-LCC-analyzer subsystem were illuminated with a halogen light source at several locations while applying different voltages to the cell. The transmitted light was measured at those locations and voltages, at a desired wavelength range. Subsequently, the ST was calculated and modeled at those locations, voltages and wavelength range, then estimated at unmeasured locations. As our research was primarily motivated to calibrate the CS-MUSI camera, the presented simulation and experiment demonstrate spatial estimation results for a relatively-thick LCC (50 μm). It should be noted that the presented method could be applied to any LCC regardless of its thickness. The proposed procedure provides an accurate description of the spatial dependency of a LCC and its ST, and can be used as a calibration method for the CS-MUSI camera or other imaging systems that exploit spectral modulators [27,28].
